# A Treasurer's Valuable Suggestions
*From an address to the Winchester and District Trades and Labour Council by Leonard Keyser, Esq., Hon. Treasurer.


**Published:** 1920-07-17

**Authors:** 


### ROYAL HAMPSHIRE COUNTY HOSPITAL.

### A Treasurer's Valuable Suggestions.*

The matter I wish to discuss with you to-night is one of very great interest to the whole community and of the very highest importance. Such being the case, I feel certain that you will give me a patient hearing, and, having heard me, I shall receive the benefit of your advice which I am seeking.

The County Hospital came into being 182 years ago, and has continuously ministered to the wants of the community during that period. I have been connected with its management for fifteen years, and for the last seven years have had the honour of acting as Hon. Treasurer. I claim, therefore, to be in a position to speak to you with some knowledge as to the work that that hospital has done and is doing. In the first place, it is a progressive institution, and as medical and surgical science has developed, so has it kept pace with all the advances which have occurred. It is now splendidly equipped, its medical and surgical staff is second to none, and we are justly proud of the devotion to their duties of matron, sisters, and nurses attached to the hospital. If you want to learn all about the hospital come and see it for yourselves. I hereby offer you a cordial invitation to pay the hospital a visit of inspection with or without notice. We will show you that when illness, serious illness or an accident, overtakes a wage-earner that he will receive at the hospital treatment, care, and attention which cannot be commanded in anybody's private house, even though the owner of that house be one of the richest in the land.

I want to impress upon you that this hospital has been built, equipped, and maintained entirely by voluntary contributions, and that the great bulk of the very large sum which it has cost has been given by those who, however ill they were, would never benefit by it. To maintain a hospital has always been a costly proceeding; to have the best of everything—and the sick need the best if they are to be cured—you must spend money. Up to now, owing to the generosity of many and the princely donations of a few, we have been able to keep the hospital going and to progress as I have stated; but now we are faced with a crisis, owing to the huge increase in the cost of everything needful to maintain a hospital. To give you one example, there has been an increase in salaries and wages of £3,659 5s. 4d. comparing 1919 with 1913. We have no intention of running the hospital on cheap and inefficient labour, and we should be foolish to expect that our nursing staff would be up to the highest standard unless we paid the highest rate of salaries. The result is this, that the hospital is now in debt to the amount of £6,000, and it requires an additional income of at least from £3,000 to £4,000 per annum to meet its ordinary expenditure. Unless these financial difficulties be overcome we must close some of the wards.

I have in my mind a solution of our difficulties, and that is enough for me. I want to go to the rich and to tell them of the state of affairs and ask them to find us the £6,000 to pay off the bankers' overdraft; but when I go to them I know what they will say (it has been said to me already), what good will it do you to liquidate this debt. Your expenditure exceeds your income from £3,000 to £4,000 a year; so if we pay off this debt for you in a very short time you will come to us again for more. I want an answer to that, and the answer I want to give them is this. The hospital is for the benefit of the wage-earners, men and women. If you will do your part they will do theirs; they (the wage-earners) are going to undertake to find the extra income which is required.

Now, gentlemen, let me tell you that the hospital management appointed a few of us to go into the question of money. We sat many times, we heard much evidence. The question before us was whether patients using the hospital should pay for the benefits they receive; that payment should be compulsory. To that I was opposed. I arrived at my opinion because it was borne in on me that when a man or woman was ill then was he least able to find the money. I expressed the view that if we went to the people and said, *if you will support us when you are well we will care for you when you are sick*, that we would get the response we desired.

The solution, then, is that every wage-earner should subscribe by way of insurance a certain sum per week—say, 1d. for every £1 he earns—or a flat rate of 2d. that will produce a very large sum—say, £2,000 a year. Then I am quite aware that there are many who use the hospital who are not wage-earners and who can pay towards their maintenance; the hospital authorities must and will exact payment from these.

I propose that in every firm all employees shall be asked to contribute weekly, a card index be kept of their names, and the amount of their contributions recorded on their cards. Will you help me? Will you form a Committee to carry this scheme out and save the hospital? The proposal I put forward, if approved and started, is going to give me an enormous amount of work, and if it has your endorsement I will carry it through. I will not grudge either the time or the work if you will not grudge your twopences and threepences, or even your fourpences and sixpences.

Gentlemen, the hospital is for your benefit; it belongs to the people, is for the people, should be supported by the people, and in so far as that support is afforded, so should the people themselves share in the management.

A discussion ensued, and it was unanimously resolved: "That a Committee of this Trades and Labour Council be formed to assist the hospital authorities."

* From an address to the Winchester and District Trades and Labour Council by. Leonard Keyser, Esq., Hon. Treasurer.

